# Patterns and predictors of opioid dispensing among older cancer patients from 2008 to 2015

**DOI:** 10.1002/cam4.70211

**Published:** 2024-10-23

**Authors:** Yingxi Chen, Yei‐Eun Shin, Susan Spillane, Meredith S. Shiels, Anna E. Coghill, Lindsey Enewold, Ruth M. Pfeiffer, Neal D. Freedman

**Affiliations:** ^1^ Division of Cancer Epidemiology and Genetics National Cancer Institute Rockville Maryland USA; ^2^ Department of Statistics Seoul National University Seoul South Korea; ^3^ College of Liberal Studies Seoul National University Seoul South Korea; ^4^ Cancer Epidemiology Program, Moffitt Cancer Center Tampa USA; ^5^ Division of Cancer Control and Population Sciences National Cancer Institute Rockville Maryland USA

**Keywords:** cancer patients, opioid medication, predictors

## Abstract

**Background:**

Understanding factors associated with opioid dispensing in cancer patients is important for developing tailored guidelines and ensuring equitable access to pain management. We examined patterns and predictors of opioid dispensing among older cancer patients from 2008 to 2015.

**Methods:**

We analyzed data from the Surveillance, Epidemiology, and End Results (SEER) database linked to Medicare claims. We included the most common cancer types among patients aged 66–95 years. Opioids dispensed within 30 days before and 120 days after cancer diagnosis were assessed. We used logistic regression models to examine trends, adjusted odds ratios (aORs), and 95% confidence intervals (CIs) for opioid dispensing, considering patient demographics, geography, cancer stage, comorbidities, and treatment options. Models were stratified by sex.

**Results:**

A total of 211,759 cancer patients aged 66–95 years were included in the study. For cancers combined, non‐Hispanic Black men had a significantly lower odds of receiving opioids during the 120 days post‐diagnosis (aOR = 0.89, 95% CI = 0.84–0.94) compared to non‐Hispanic White men. Factors such as pre‐diagnosis opioid dispensing, age, geography, cancer stage, comorbidities, and type of cancer treatment were associated with opioid dispensing during the 120 days post‐diagnosis. Surgery had the strongest association, with men undergoing surgery being 4.4 times more likely to receive opioids within 120 days post‐diagnosis (aOR = 4.41, 95% CI = 4.23–4.60), while women had an odds ratio of 2.72 (95% CI = 2.62–2.83). Chemotherapy and radiotherapy were also positively associated with opioid dispensing, with less pronounced estimates.

**Conclusions:**

We observed significant variations in opioid dispensing among cancer patients aged 66‐95 years across cancer types and demographic and clinical factors.

## 1 INTRODUCTION

The opioid epidemic in the United States has prompted regulations aimed to address prescribing practices and mitigate risks, impacting access to opioids for cancer patients. Our recent findings revealed a significant decline in the annual prevalence of opioid claims among cancer patients following the 2016 Centers for Disease Control and Prevention (CDC) Guideline for Prescribing Opioids for Chronic Pain.^1^, ^2^ Although the Guideline explicitly excluded patients with active cancer, oncologists reported a widespread misinterpretation that these guidelines apply to cancer patients.^3^ To address this, the CDC updated its guideline in 2022, clarifying the exclusion of patients with cancer‐related pain.^4^ Nonetheless, there remains a pressing need to identify factors before these guidelines were introduced that are associated with opioid dispensing in cancer patients, especially during the period of heightened opioid use in order to understand the underlying patterns in opioid prescription for cancer patients.

Moreover, recent data from the United States highlight racial and ethnic disparities in opioid prescribing among cancer patients, with non‐Hispanic Black cancer patients being less likely to receive opioid prescriptions compared to non‐Hispanic White patients.^5^ While disparities in opioid prescriptions have been reported, the factors associated with opioid dispensing among cancer patients remain incompletely understood. Examining data from a period of high opioid use will provide important insights into disparities in opioid dispensing among different demographic groups across various types of cancer.

The aims of this study were to (1) describe the pattern of opioid dispensing ≤30 days pre‐diagnosis and ≤120 days post‐diagnosis in cancer patients and (2) to identify the demographic and clinical factors associated with opioid dispensing during the ≤120 days post‐diagnosis period. We analyzed data from 2008 to 2015, the period prior to the 2016 CDC guidelines that led to a significant reduction in opioid use among cancer patients. This allows us to capture prescribing practices prior to the significant changes induced by these guidelines. In addition, our study builds on previous research^5^ by including data from 2014 and 2015 and by analyzing a broader range of cancer sites. We consider a variety of factors, such as patient demographics, overall health status, geographic location, treatment options, and healthcare services, to provide a more comprehensive analysis of opioid dispensing patterns immediately after a cancer diagnosis. Understanding these factors will provide a comprehensive understanding of the complex dynamics surrounding opioid dispensing in the early stages after a cancer diagnosis and for informing the development of guidelines to support patient care.

## 2 METHODS

### 2.1 Data and study population

Cancer patients were selected from the Surveillance, Epidemiology, and End Results (SEER) database linked to Medicare claims.^6^ The SEER data is composed of cancer registries covering various geographic locations, collectively in this analysis representing 28% of cancer cases in the United States The SEER registries gather data on newly diagnosed cancer cases, including information on clinical characteristics, treatment, and patient demographics. Medicare, a federally funded health insurance program mainly available to individuals ≥65 years in the United States, provides clinical and administrative data including diagnoses, hospitalizations, emergency department visits, outpatient encounters, and prescription drug information. The combined SEER‐Medicare dataset facilitates the longitudinal evaluation of care patterns for elderly cancer patients.

In this study, we included the most common sites of cancer among both men and women in the United States population aged 66–95 years: colon and rectum, female breast, kidney and renal pelvis, lung and bronchus, melanomas of the skin, prostate, and urinary bladder. These seven sites were identified from a recent case–control study examining risk factors associated with the incidence of cancers within the SEER‐Medicare population.^7^ We included cancer patients diagnosed between February 2008 and June 2015, with stage 1,2,3,4 (non‐bladder) and stage 0,1,2,3,4 (bladder) disease. We included in situ bladder cancers as it is standard practice in cancer surveillance for bladder cancer, ensuring consistency and comparability with other cancer studies and surveillance reports.^8^

Patients were required to have continuous enrollment in fee‐for‐service Medicare Part A, B, and D during 12 months prior to the month of diagnosis through 120 days post‐diagnosis or until death, whichever came first. A year of enrollment was required prior to diagnosis to determine comorbidity at diagnosis. We excluded patients with more than one primary tumor, missing data on the month of diagnosis, and patients diagnosed at autopsy.

### 2.2 Definitions and study covariates

The opioid drugs included in the analysis encompass a range of medications, including opioid and acetaminophen/NSAID combinations, hydrocodone, oxycodone, tramadol, codeine, fentanyl, propoxyphene, morphine, and hydromorphone. Opioid dispensing was identified using the FDA National Drug Code (NDC), which comprehensively covers various drugs and formulations of opioids (Table S1). Opioid dispensing at the time of cancer diagnosis was defined as having opioids dispensed between 30‐day prior to cancer diagnosis and the date of diagnosis. Post‐diagnosis opioid dispensing was defined as opioid dispensing between the date of cancer diagnosis and 120‐day after cancer diagnosis. Opioid dispensing data were ascertained from Medicare Part D data.

We used SEER registry data to ascertain baseline patient demographic, tumor data, and treatment data on surgery and radiotherapy, and we used physician claims data from Medicare to obtain data on opioid dispensing, systemic cancer treatment and comorbidities.^9^ For assessing patient comorbidities, we calculated the NCI‐adapted Charlson Comorbidity Index,^10^ which provides a standardized approach for quantifying the non‐cancer comorbidity burden based on medical claims in the year prior to a patient's cancer diagnosis.

### 2.3 Statistical analyses

We estimated the proportion of opioid dispensing among cancer patients in the periods 30 days before and 120 days after cancer diagnosis. We then examined the predictors for opioid dispensing within 120 days post‐diagnosis (any dispensing yes/no). We first assessed the trend over calendar time on the prevalence of opioid dispensing by fitting logistic regression models with opioid dispensing as the outcome in the respective time period. In these models, the calendar year (2008–2015) was used as an ordinal variable. We then estimated adjusted odds ratios (aORs) and 95% confidence intervals (CIs) from models that additionally included cancer type (colon and rectum, female breast, kidney and renal pelvis, lung and bronchus, melanomas of the skin, prostate, and urinary bladder; urinary bladder cancer was arbitrarily assigned as the reference group), age at diagnosis group (<70, 70–74, 75–79, 80–95), stage (0–4 for urinary bladder cancer; 1–4 for all other cancers), race and ethnicity (Hispanic/Latino, non‐Hispanic Asian/Pacific Islander, non‐Hispanic Black, non‐Hispanic White, and non‐Hispanic others and unknown racial groups), SEER region (Western region, Northeastern region, North‐central region, Southern region),^11^ Charlson comorbidity score (0,1, and 2+), number of physician visits (in quantiles; based on visits that occurred in 6–12 month prior to diagnosis) and number of physician visits (in quantiles; based on visits that occurred within 6 months prior to diagnosis). We also additionally adjusted for opioid dispensing in the period 30 days pre‐diagnosis (any dispensing yes/no) and cancer treatments that were recorded within 120 days post‐diagnosis (yes/no for radiotherapy, chemotherapy, and surgery, respectively). Due to significant interactions between sex and key variables (*p*_interaction <0.05), all analyses were stratified by sex.

## 3 RESULTS

### 3.1 Patient characteristics

Between February 2008 and June 2015, records of 211,759 cancer patients aged 66–95 years contributed to the study. The mean age was 76 years (standard deviation [SD]: 6.8) and 50.5% (*N* = 106,991) were female. About 79.7% (*N* = 168,731) of the patients were non‐Hispanic White, 5.0% (*N* = 10,496) were non‐Hispanic Black, 6.0% (12,785) were Hispanic/Latino, and 7.9% (*N* = 16,804) were non‐Hispanic Asian/Pacific Islanders. Over 40% (*N* = 85,526) of the patients were in the Western region, followed by the Southern region at 26.7%, (*N* = 56,610), the Northcentral region at 20.6% (43,633), and the Northeastern region at 12.3% (*N* = 25,990) (Table 1).

**TABLE 1:** Characteristics of cancer patients, SEER‐Medicare PartD, 2008–2015.

Characteristics		Men		Women		Total	
		*N* = 104,768	%	N = 106,991	%	*N* = 211,759	%
Year	2008	12,465	11.9	12,381	11.6	24,846	11.7
	2009	13,625	13.0	13,724	12.8	27,349	12.9
	2010	13,490	12.9	13,465	12.6	26,955	12.7
	2011	13,688	13.1	13,605	12.7	27,293	12.9
	2012	13,221	12.6	14,063	13.1	27,284	12.9
	2013	14,073	13.4	14,848	13.9	28,921	13.7
	2014	15,719	15.0	16,582	15.5	32,301	15.3
	2015	8487	8.1	8323	7.8	16,810	7.9
Age group	66–70	24,636	23.5	20,373	19.0	45,009	21.3
	70–74	31,825	30.4	26,577	24.8	58,402	27.6
	75–79	22,958	21.9	23,306	21.8	46,264	21.8
	80–95	25,349	24.2	36,735	34.3	62,084	29.3
Race and ethnicity	NHW	82,133	78.4	86,598	80.9	168,731	79.7
	API	8078	7.7	8726	8.2	16,804	7.9
	Hispanic	6985	6.7	5800	5.4	12,785	6.0
	NHB	5596	5.3	4900	4.6	10,496	5.0
	Non‐Hispanic other/unknown	1976	1.9	967	0.9	2943	1.4
Region*	Western	43,971	42.0	41,555	38.8	85,526	40.4
	Northcentral	20,265	19.3	23,368	21.8	43,633	20.6
	Northeastern	12,589	12.0	13,401	12.5	25,990	12.3
	Southern	27,943	26.7	28,667	26.8	56,610	26.7
Cancer type	Colon and rectum	13,099	12.5	18,160	17.0	31,259	14.8
	Female breast			44,544	41.6	44,544	21.0
	Kidney and renal pelvis	5247	5.0	4637	4.3	9884	4.7
	Lung and bronchus	23,443	22.4	28,814	26.9	52,257	24.7
	Melanomas of the skin	7020	6.7	5038	4.7	12,058	5.7
	Prostate	42,523	40.6			42,523	20.1
	Urinary bladder	13,436	12.8	5798	5.4	19,234	9.1
Stage	0 (urinary bladder)	7423	7.1	2946	2.8	10,369	4.9
	1	19,915	19.0	43,547	40.7	63,462	30.0
	2	43,535	41.6	22,963	21.5	66,498	31.4
	3	14,091	13.4	17,303	16.2	31,394	14.8
	4	19,804	18.9	20,232	18.9	40,036	18.9
Charlson's score	0	42,655	40.7	43,863	41.0	86,518	40.9
	1	26,558	25.3	28,630	26.8	55,188	26.1
	2+	33,675	32.1	33,344	31.2	67,019	31.6
	Missing	1880	1.8	1154	1.1	3034	1.4
Number of physician visits within 6 months prior to diagnosis	0–1	26,126	24.9	26,484	24.8	52,610	24.8
	2–3	26,850	25.6	26,632	24.9	53,482	25.3
	4–6	25,894	24.7	26,107	24.4	52,001	24.6
	7–129	25,898	24.7	27,768	26.0	53,666	25.3
Number of physician visits in 6–12 months prior to diagnosis	0–1	27,184	25.9	24,340	22.7	51,524	24.3
	2–3	25,563	24.4	25,787	24.1	51,350	24.2
	4–7	29,718	28.4	31,351	29.3	61,069	28.8
	8–183	22,303	21.3	25,513	23.8	47,816	22.6
Radiotherapy within 120‐day post‐diagnosis	No	84,769	80.9	84,858	79.3	169,627	80.1
	Yes	19,999	19.1	22,133	20.7	42,132	19.9
Chemotherapy within 120‐day post‐diagnosis	No	87,561	83.6	85,868	80.3	173,429	81.9
	Yes	17,207	16.4	21,123	19.7	38,330	18.1
Surgery within 120‐day post‐diagnosis	No	62,984	60.1	38,940	36.4	101,924	48.1
	Yes	41,784	39.9	68,051	63.6	109,835	51.9

* Western: Hawaii, New Mexico, Seattle‐Puget Sound, Utah, San Francisco‐Oakland, San Jose‐Monterey, Los Angeles, Greater California, Idaho, Arizona, Alaska, Cherokee Nation; Northcentral: Iowa, Illinois; Northeastern: Connecticut, Massachusetts, New Jersey, New York; Southern: Kentucky, Louisiana, Atlanta, Rural Georgia, Texas.

Cancers of the lung and bronchus accounted for 24.7% (*N* = 52,257) of the study population, followed by female breast cancer (21.0%, *N* = 44,544), prostate cancer (20.1%, *N* = 42,523), cancers of the colon and rectum (14.8%, *N* = 31,259), urinary bladder cancer (9.1%, *N* = 19,234), melanomas of the skin (5.7%, *N* = 12,058), and cancers of the kidney and renal pelvis (4.7%, *N* = 9884). Over 60% of the cancer patients were stage 2 or below at the time of diagnosis, and 40.9% had a Charlson comorbid index of 0, that is such patients were not identified by this index as having comorbidities. Over 50% of patients received surgery within 120 days post‐diagnosis, about 20% received radiotherapy and 18% received chemotherapy (Table 1).

### 3.2 Opioids dispensing in the 30‐day period pre‐diagnosis and the 120‐day period post‐diagnosis

Overall, 16.4% (*N* = 34,831) of the patients had a record of opioid dispensing within the 30‐day period prior to cancer diagnosis. Patients diagnosed with lung cancer had the highest proportion (24.3%, *N* = 12,695), followed by patients with kidney cancer (21.8%, *N* = 2155), urinary bladder cancer (14.7%, *N* = 2832), colorectal cancer (14.3%, *N* = 4461), female breast cancer (14.2%, *N* = 6314), melanoma (11.8%, *N* = 1418), and prostate cancer (11.7%, *N* = 4956; Table 2).

**TABLE 2:** Opioid dispensing in the 30‐day period pre‐ diagnosis and the 120‐day period post‐diagnosis, by cancer sites SEER‐Medicare PartD, 2008–2015.

	Colon and rectum		Female breast		Kidney/renal pelvis		Lung and bronchus		Melanomas of the skin		Prostate		Urinary bladder	
Total patients, *N* (%)	31,259	(100)	44,544	(100)	9884	(100)	52,257	(100)	12,058	(100)	42,523	(100)	19,234	(100)
30 days pre‐diagnosis, *N* (%)	4461	(14.3)	6314	(14.2)	2155	(21.8)	12,695	(24.3)	1418	(11.8)	4956	(11.7)	2832	(14.7)
120‐days post‐diagnosis, *N* (%)	17,546	(56.1)	33,354	(74.9)	6941	(70.2)	33,159	(63.5)	6795	(56.4)	16,900	(39.7)	11,874	(61.7)

The proportion of opioid dispensing dramatically increased within 120 days post‐diagnosis compared to the pre‐diagnosis period, by nearly three‐fold (post‐diagnosis dispensing 59.8%, *N* = 126,569). Female breast cancer patients had an over four‐time increase in dispensing (74.9%, *N* = 33,354), followed by patients with melanoma (56.4%, *N* = 6795), urinary bladder cancer (61.7%, *N* = 11,874), colorectal cancer (56.1%, *N* = 17,546), prostate cancer (39.7%, *N* = 16,900), kidney cancer (70.2%, *N* = 6941), and lung cancer (63.5%, *N* = 33,159) (Table 2).

### 3.3 Predictors of opioid dispensing during the 120‐day post‐diagnosis period

Table 3 shows the predictors of opioid dispensing within the 120‐day post‐diagnosis period, estimated separately for men and women using sex‐stratified models. Pre‐diagnosis opioid dispensing was significantly associated with post‐diagnosis opioid dispensing (aOR and 95% CI: in women 1.37, 1.35–1.38; in men 1.40, 1.39–1.41). Opioid dispensing also decreased significantly with increasing age (*p* < 0.001). Compared to non‐Hispanic White men with cancer, non‐Hispanic Black men with cancer were significantly less likely to receive opioids (0.89, 0.84–0.94). We also observed significant geographic variation in post‐diagnosis opioid dispensing. For example, compared to patients in the Western region, patients residing in the Southern region were 17% (in women, 1.17, 1.12–1.21) and 35% (in men, 1.35, 1.30–1.40) more likely to receive opioids, while patients in the Northcentral and Northeastern region were 10% and 30% less likely to receive opioids in both women and men. We observed significant increasing calendar time trends in relation to opioid dispensing in both women (*p* = 0.02) and men (*p* = 0.001). Compared to the year 2008, patients diagnosed in later years were more likely to receive opioids, except for the year 2015 (women, 0.97, 0.91–1.04; men, 0.97, 0.91–1.04). Moreover, the number of physician visits prior to diagnosis was also associated with opioid dispensing, though the direction and magnitude of association varied by time of visit across sex (Table 3). Furthermore, we also observed sex differences in opioid dispensing in relation to stage of disease. For example, compared to stage 1 cancer, men with stage 4 cancer were 2.3 times more likely (2.30, 2.18–2.43) to receive opioid within 120 days post‐diagnosis while women with stage 4 cancer were about 1.4 times more likely (1.37, 1.31–1.44) to receive opioids.

**TABLE 3:** Logistic regression analysess of opioid dispensing within 120‐day post‐diagnosis, 2008–2015.

Characteristics		Univariable analysis				Multivariable analysis			
		Men		Women		Men		Women	
		OR (95% CI)	*p*‐value	OR (95% CI)	*p*‐value	Adjusted OR (95% CI)	*p*‐value	Adjusted OR (95% CI)	*p*‐value
Intercept		–	–	–	–	0.38 (0.34–0.42)	–	0.77 (0.68–0.86)	–
30‐day pre‐diagnosis opioids dispensing, yes		0.80 (0.79–0.81)	<0.001	1.49 (1.47–1.51)	<0.001	1.40 (1.39–1.41)	<0.001	1.37 (1.35–1.38)	<0.001
Age group, years	66–70	Reference	<0.001	Reference	<0.001	Reference	<0.001	Reference	<0.001
	70–74	0.86 (0.83–0.89)		0.85 (0.82–0.89)		0.89 (0.86–0.93)		0.89 (0.85–0.93)	
	75–79	0.73 (0.71–0.76)		0.65 (0.63–0.68)		0.74 (0.71–0.77)		0.73 (0.69–0.76)	
	80–95	0.61 (0.59–0.63)		0.38 (0.37–0.40)		0.58 (0.55–0.60)		0.48 (0.46–0.50)	
Race and Ethnicity	NHW	Reference	<0.001	Reference	<0.001	Reference	<0.001	Reference	<0.001
	API	0.82 (0.77–0.86)				0.95 (0.89–1.01)		0.93 (0.87–1.00)	
	Hispanic	0.96 (0.91–1.01)				1.07 (1.01–1.13)		1.07 (1.00–1.14)	
	NHB	0.94 (0.90–0.98)				0.89 (0.84–0.94)		0.99 (0.94–1.05)	
	Non‐Hispanic other/unknown	0.51 (0.47–0.56)				0.69 (0.62–0.76)		0.79 (0.68–0.91)	
Region	Western	Reference	<0.001	Reference	<0.001	Reference	<0.001	Reference	<0.001
	Northcentral	0.93 (0.89–0.97)		0.90 (0.86–0.94)		0.88 (0.84–0.93)		0.92 (0.87–0.96)	
	Northeastern	0.65 (0.63–0.67)		0.62 (0.60–0.64)		0.67 (0.64–0.69)		0.71 (0.68–0.73)	
	Southern	1.42 (1.38–1.47)		1.25 (1.20–1.29)		1.35 (1.30–1.40)		1.17 (1.12–1.21)	
Year	2008	Reference	0.07	Reference	<0.001	Reference	0.001	Reference	0.019
	2009	1.09 (1.03–1.14)		1.06 (1.00–1.11)		1.07 (1.01–1.13)		1.05 (0.99–1.11)	
	2010	1.10 (1.04–1.15)		1.07 (1.02–1.13)		1.05 (0.99–1.11)		1.08 (1.02–1.14)	
	2011	1.13 (1.08–1.19)		1.07 (1.02–1.13)		1.08 (1.02–1.14)		1.06 (1.00–1.12)	
	2012	1.18 (1.13–1.24)		1.09 (1.03–1.14)		1.09 (1.03–1.15)		1.06 (1.00–1.12)	
	2013	1.17 (1.12–1.23)		1.07 (1.02–1.13)		1.08 (1.02–1.14)		1.06 (1.00–1.12)	
	2014	1.15 (1.10–1.21)		1.08 (1.03–1.14)		1.07 (1.01–1.13)		1.06 (1.01–1.12)	
	2015	1.10 (1.04–1.16)		1.04 (0.99–1.11)		0.97 (0.91–1.04)		0.97 (0.91–1.04)	
Cancer type	Colon and Rectum	0.77 (0.74–0.81)	<0.001	0.81 (0.77–0.86)	<0.001	0.68 (0.63–0.73)	<0.001	0.74 (0.67–0.81)	<0.001
	Female Breast	–		1.87 (1.76–1.98)		–		1.75 (1.61–1.92)	
	Kidney and Renal Pelvis	1.36 (1.27–1.46)		1.60 (1.47–1.73)		1.66 (1.52–1.82)		1.63 (1.46–1.83)	
	Lung and Bronchus	0.97 (0.93–1.02)		1.18 (1.11–1.25)		1.48 (1.37–1.59)		1.41 (1.28–1.54)	
	Melanomas of the Skin	0.79 (0.74–0.84)		0.82 (0.76–0.89)		0.87 (0.81–0.95)		0.76 (0.68–0.84)	
	Prostate	0.41 (0.39–0.42)		–		0.95 (0.88–1.03)		–	
	Urinary Bladder	Reference		Reference		Reference		Reference	
Stage (seven sites combined)	0	1.08 (1.03–1.14)	<0.001	0.58 (0.54–0.62)	<0.001	0.88 (0.81–0.95)	<0.001	0.68 (0.60–0.76)	<0.001
	1	Reference		Reference		Reference		Reference	
	2	0.52 (0.50–0.53)		0.96 (0.93–1.00)		1.13 (1.07–1.19)		1.04 (1.00–1.09)	
	3	1.17 (1.12–1.22)		0.79 (0.76–0.82)		1.53 (1.45–1.62)		1.06 (1.01–1.11)	
	4	1.19 (1.14–1.24)		0.79 (0.77–0.82)		2.30 (2.18–2.43)		1.37 (1.31–1.44)	
Charlson's score	0	Reference	0.006	Reference	<0.001	Reference	<0.001	Reference	<0.001
	1	1.20 (1.16–1.23)		0.97 (0.94–1.00)		1.02 (0.99–1.06)		0.97 (0.94–1.01)	
	2+	1.25 (1.21–1.28)		0.89 (0.87–0.92)		0.92 (0.88–0.95)		0.86 (0.82–0.89)	
Number of physician visits within 6 months prior to diagnosis (in quantile)	0–1	Reference	<0.001	Reference	<0.001	Reference	0.004	Reference	0.34
	2–3	1.09 (1.05–1.12)		1.07 (1.04–1.11)		1.06 (1.02–1.10)		1.04 (0.99–1.08)	
	4–6	1.16 (1.12–1.21)		1.07 (1.04–1.11)		1.08 (1.03–1.13)		1.01 (0.96–1.05)	
	7–129	1.33 (1.29–1.38)		1.12 (1.08–1.16)		1.06 (1.01–1.11)		1.02 (0.97–1.07)	
Number of physician visits in 6–12 months prior to diagnosis (in quantile)	0–1	Reference	0.054	Reference	<0.001	Reference	<0.001	Reference	<0.001
	2–3	1.07 (1.03–1.11)		1.10 (1.06–1.14)		0.96 (0.92–1.00)		1.05 (1.01–1.10)	
	4–7	1.13 (1.09–1.16)		1.13 (1.09–1.17)		0.94 (0.90–0.98)		1.01 (0.97–1.06)	
	8–183	1.27 (1.22–1.31)		1.14 (1.10–1.18)		0.88 (0.84–0.93)		0.90 (0.85–0.95)	
Radiotherapy within 120‐day after diagnosis, yes		0.89 (0.86–0.92)	<0.001	1.40 (1.36–1.45)	<0.001	1.15 (1.11–1.19)	<0.001	1.21 (1.17–1.26)	<0.001
Chemotherapy within 120‐day after diagnosis, yes		2.16 (2.09–2.24)	<0.001	1.43 (1.38–1.48)	<0.001	1.58 (1.51–1.65)	<0.001	1.46 (1.41–1.52)	<0.001
Surgery within 120‐day after diagnosis, yes		2.58 (2.51–2.64)	<0.001	1.77 (1.72–1.82)	<0.001	4.41 (4.23–4.60)	<0.001	2.72 (2.62–2.83)	<0.001

Opioid dispensing also varied by type of cancer diagnosis. Compared to urinary bladder cancer patients, patients with kidney cancer (women, 1.63, 1.46–1.83; men, 1.66, 1.52–1.82), lung cancer (women, 1.41, 1.28–1.54; men, 1.48, 1.37–1.59), and female breast cancer (1.75, 1.61–1.92) were more likely to receive opioids within 120 days post‐diagnosis, while patients with colorectal cancer (women, 0.74, 0.67–0.81; men, 0.68, 0.63–0.73), melanoma (women, 0.76, 0.68–0.84; men, 0.87, 0.81–0.95) were less likely to receive opioids. Additionally, patients diagnosed at more advanced stages and with a lower Charlson comorbid index were more likely to receive opioids. Moreover, among men there was a significant association between the number of physician visits within 6 months prior to diagnosis and opioids dispensed ≤120 days post‐diagnosis (*p =* 0.004), whereas there was no significant association among women (*p =* 0.34:). Patients receiving surgery within 120 days post‐diagnosis had an increased odds of receiving opioids compared to those who did not undergo surgery (in women, 2.72, 2.62–2.83; in men, 4.41, 4.23–4.60). Radiotherapy and chemotherapy within 120 days post‐diagnosis were also positively associated with opioids dispensing compared to those who did not receive radiotherapy or chemotherapy, respectively, though the associations were less prominent (Table 3).

## 4 DISCUSSION

This population‐based study of older adults revealed that between 2008 and 2015, 16.4% of cancer patients had an opioid dispensing record ≤30 days pre‐diagnosis, which rose to 59.8% within ≤120 days post‐diagnosis. Notably, the increase in opioid dispensing varied across cancer types. For examples, female breast cancer patients had an over four‐fold increase in dispensing from 14.2% within 30 days pre‐diagnosis to 74.9% within 120 days post‐diagnosis, while among lung cancer patients it was from 24.3% within 30 days pre‐diagnosis to 63.5% within 120 days post‐diagnosis. Furthermore, factors such as pre‐diagnosis opioid dispensing, age, race and ethnicity, stage, comorbidities, and types of cancer treatment were significantly associated with opioid dispensing ≤120 days post‐diagnosis. Understanding opioid dispensing during a period of high opioid use provides a critical contextual background for comprehending broader dispensing trends.

We found a concerning racial disparity in opioid dispensing among cancer patients, specifically for non‐Hispanic Black men, who were significantly less likely to receive opioids ≤120 days post‐diagnosis compared to other groups. Previous data suggest that Black patients are more likely to experience underdiagnosed pain^12^, ^13^, ^14^ and are less likely to receive sufficient pain medication.^15^ A recent United States study examining 2007–2019 trends in opioid prescription fills near the end of life found that older Black patients with advanced cancer were less likely to receive opioid medication for pain relief in the last weeks of life than White patients.^16^ Despite national efforts to address racial disparities in assessment and treatment of cancer‐related pain,^17^ Black men still are less likely to receive opioids following a cancer diagnosis. This persistent disparity raises important questions about equitable access to pain management and highlights the need to address potential racial biases in healthcare settings.

Our findings indicate that pre‐diagnosis opioid dispensing was an important predictor for post‐diagnosis opioid dispensing. Opioid use prior to a cancer diagnosis increases the likelihood of long‐term use following the diagnosis.^18^ This underscores the importance of understanding the patterns and factors influencing opioid use before a cancer diagnosis. A recent population‐based study found considerable heterogeneities in opioid use before and after cancer diagnosis, suggesting that noncancer factors drive a significant proportions of post‐diagnosis opioid use.^19^ Pre‐diagnosis opioid use may reflect underlying chronic pain conditions or other health issues that necessitate opioid therapy, which could influence pain management strategies and overall treatment outcomes in cancer patients. These results highlight the need for comprehensive pain management approaches that consider pre‐existing opioid use and its potential impact on post‐diagnosis care and survivorship.

Furthermore, our results highlight a notable sex difference in opioid dispensing among cancer patients. When compared to stage 0 cancer, men with stage 4 cancer were 2.3 times more likely to receive opioids ≤120 days post‐diagnosis, while the odds ratio for females was 1.4. Moreover, men had a significant association between the number of physician visits within 6 months prior to diagnosis and opioid dispensing ≤120 days post‐diagnosis, whereas women did not show a significant association. It is unclear whether these sex‐specific differences may be attributed to physician bias, clinical indications, or healthcare access.

We also found that opioid dispensing varied across treatment options in cancer patients. Compared to patients who did not receive surgery, patients receiving surgery within ≤120 days post‐diagnosis was the strongest predictor for opioid dispensing ≤120‐day post‐diagnosis. The effect of radiotherapy on opioid dispensing was less pronounced, likely due to its pain relief potential in multiple clinical scenarios.^20^ Notably, we observed that cancer patients with comorbidities were less likely to receive opioids compared to those without comorbid conditions. Similarly, opioid dispensing also decreased significantly with increasing age. This may be related to individual risk–benefit assessments where among patients with comorbidities, healthcare providers weigh the potential benefits of pain relief against the side effects of opioids, considering factors such as age, polypharmacy use, overall health, and treatment goals.^21^, ^22^, ^23^

Our study has several limitations. First, as we exclusively analyzed SEER‐Medicare administrative data, the trends observed may not represent opioid dispensing patterns in younger patients or other specific patient groups. However, the elderly form the largest proportion of all US cancer patients, making our study highly relevant to a substantial portion of the population. Second, the 30‐day period pre‐diagnosis may not capture all opioids dispensing at the time of diagnosis. Nonetheless, the intention was to identify cases of chronic opioid use pre‐diagnosis, which should be reasonably captured within the 30‐day window. Third, about 1.4% of our study population was captured as non‐Hispanic others or unknown racial groups. This group may include individuals with diverse racial and ethnic backgrounds that were not adequately captured by the available categories. Therefore, the findings related to this subgroup might not be as generalizable as those for more well‐represented racial and ethnic groups. Fourth, it is possible that patients received opioids outside of Medicare, potentially leading to an underestimation of dispensing or use. In addition, we were unable to assess the appropriateness of opioid dispensing, limiting our ability to thoroughly understand and define the observed disparities. Moreover, it is worth noting that the capture of radiotherapy treatment relied on data from the SEER registry, which could potentially be subject to under‐ascertainment. Additionally, while this study focuses on broad patterns of opioid prescription among older cancer patients, detailed multivariate analyses of cancer type, stage, and treatment modalities were not feasible due to data limitations and the scope of this analysis. Future research with more comprehensive data could provide further insights into these factors. It is also important to note that opioids prescribed in hospice programs may not be fully captured in our data. Consequently, our study may not capture all opioid dispensing, especially those related to end‐of‐life care. This limitation could affect the completeness of our results. Last but not the least, due to the nature of the study design, we were unable to differentiate between opioid dispensing and actual consumption. Nonetheless, our study contributes important real‐world evidence on opioids dispensing in cancer patients, providing critical population level data to inform clinical practice.

## 5 CONCLUSION

Our study provides insights into opioid dispensing patterns among older cancer patients, highlighting notable variation across cancer types and demographic and clinical factors. Understanding opioid dispensing dynamics after a cancer diagnosis is crucial for clinical practice and patient care. Future research is needed to investigate disparities in the duration and dosage of opioid therapy among cancer patients. Such studies are essential for understanding the full scope of opioid use and for developing tailored guidelines to optimize opioid use in older cancer patients, thereby improving patient care.

## AUTHOR CONTRIBUTIONS

**Yingxi Chen:** Conceptualization (supporting); funding acquisition (supporting); investigation (supporting); methodology (supporting); writing – original draft (lead); writing – review and editing (lead). **Yei‐Eun Shin:** Formal analysis (lead); methodology (equal); writing – review and editing (supporting). **Susan Spillane:** Conceptualization (supporting); data curation (equal); writing – review and editing (supporting). **Meredith S. Shiels:** Methodology (supporting); writing – review and editing (supporting). **Anna E. Coghill:** Writing – review and editing (supporting). **Lindsey Enewold:** Methodology (supporting); writing – review and editing (supporting). **Ruth Pfeiffer:** Conceptualization (supporting); formal analysis (supporting); investigation (supporting); methodology (equal); writing – review and editing (supporting). **Neal D. Freedman:** Conceptualization (lead); data curation (equal); formal analysis (supporting); funding acquisition (lead); methodology (supporting); writing – review and editing (supporting).

## FUNDING INFORMATION

This study was funded by the NIH Intramural Research Program.

## CONFLICT OF INTEREST STATEMENT

The authors declare no conflicts of interest.

## PRECIS

The study analyzed 211,759 cancer patients aged 66–95 and found that non‐Hispanic Black men had significantly lower odds of receiving opioids after cancer diagnosis compared to non‐Hispanic White men. Overall, we observed variations in opioid dispensing, influenced by pre‐diagnosis opioid dispensing, age, geography, cancer stage, comorbidities, and type of cancer treatment.

## Supporting information


Table S1.

## Data Availability

SEER‐Medicare data are available upon approval at: https://healthcaredelivery.cancer.gov/seermedicare/aboutdata/program.html.
